# MAPK-dependent copper tolerance mechanisms revealed by integrated physiology and transcriptomics in peanut

**DOI:** 10.3389/fpls.2025.1667121

**Published:** 2025-10-21

**Authors:** XueFeng Bao, WeiMin Ning, ZhiQiang Gao, Mian Hu, Xuan Dong

**Affiliations:** Panxi Crop Improvement Key Laboratory of Sichuan Province, College of Agricultural Science, Xichang University, Xichang, Sichuan, China

**Keywords:** *Arachis hypogaea*, copper stress, physiological response, MAPK pathway, gene expression

## Abstract

**Introduction:**

To elucidate the physiological and molecular responses of peanut (*Arachis hypogaea L* L. c.v. ‘*Haihua No. 1*’) to copper stress, this study aimed to investigate the changes in root morphology, ion content, oxidative stress, and gene expression under copper stress conditions.

**Methods:**

Seedlings were exposed to 0 (control) or 50 mg/L CuSO₄ solution, with three biological replicates for each treatment. Root length and biomass were measured quantitatively, along with tissue contents of eight ions (K^+^, Na^+^, Mg^2+^, Ca^2+^, Fe^3+^, Mn^2+^, Cu^2+^, Zn^2+^), secondary oxidative stress indices, and activities of key antioxidant enzymes. RNA-seq and qPCR validation were performed to analyze transcriptional changes and identify specific gene-response modules in peanut seedling roots under copper stress.

**Results:**

Copper stress significantly induced the expression of *MPK4*, a key component of the *MPK4* pathway. Post-translationally, MPK4 likely phosphorylated two critical protein classes: NAC and LBD. NAC functioned as a core transcription factor, directly regulating the transcription of copper defense-related genes. LBD directly down-regulated genes associated with lateral root growth, indirectly promoting the expression of genes involved in GSH-dependent heavy metal detoxification and secondary oxidative stress (e.g., *GST* and *POD*), thereby enhancing the plant's detoxification and antioxidant capacity.

**Discussion:**

This study provides insights into the regulatory mechanisms that peanut plants employ to cope with copper stress. The findings highlight the roles of *MPK4*, NAC, and LBD in the plant's response to copper stress and suggest that these genes could be targeted in breeding programs to develop copper-tolerant peanut cultivars. The results may provide theoretical support for the development of such cultivars.

## Introduction

With the rapid acceleration of industrialization and continuing population growth, heavy-metal contamination of soils has escalated into a critical global environmental concern (Li et al., 2020). According to the Survey Bulletin on the National Soil Contamination Status (Ministry of Environmental Protection and Ministry of Land and Resources, April 2014), 16.1% of all monitored sites across China exceeded national environmental quality standards; among these non-compliant sites, 82.8% were attributed to inorganic pollutants, and copper (Cu^2+^) alone exhibited a site-exceedance rate of 2.1% (Wu et al., 2021). In China, the Southwest region constitutes a major non-ferrous metallogenic belt, and mining as well as smelting operations within this region are the dominant sources of soil contamination by Cu^2+^, Fe^3+^, Zn^2+^, and other heavy metals (Borkert and Cox, 1999). For example, soils surrounding the Dongchuan and Luchun copper mines in Yunnan Province exhibit pronounced heavy-metal enrichment, with concentrations markedly exceeding regional geochemical background values (Afzal et al., 2022). Such contamination not only suppresses local vegetation growth but also poses a potential threat to human health via trophic transfer through the food web (Demecsová et al., 2020).

Peanut (*Arachis hypogaea L.*) is cultivated worldwide and serves as a pivotal economic and oilseed crop in southwest China; its yield and quality directly determine agricultural productivity and food safety in peanut-producing regions (Kobayashi et al., 2019). Nevertheless, in Cu^2+^-contaminated soils both vegetative growth and kernel quality are severely compromised. Excess Cu^2+^ not only suppresses plant development, but also compromises the safety of peanuts destined for human consumption (Sharma et al., 2021). Therefore, elucidating the response mechanisms of peanut to Cu^2+^ stress is essential for ensuring regional agricultural sustainability and food security.

During long-term evolution, plants have developed a sophisticated network of heavy-metal stress responses, including antioxidant defense, signal transduction, and ion-homeostatic regulation to adapt to their surrounding geochemical environments (Cai et al., 2023). Under metal stress, excessive reactive oxygen species (ROS) are scavenged through the activation of antioxidant enzymes such as superoxide dismutase (SOD), glutathione peroxidase (GPX), and catalase (CAT) (Wang et al., 2021). Concomitantly, plants maintain intracellular metal homeostasis via finely tuned ion-balance mechanisms; for instance, under Fe^3+^ deficiency, the expression of FERRIC REDUCTION OXIDASE 2 (*FRO2*) and IRON-REGULATED TRANSPORTER 1 (*IRT1*) is up-regulated to enhance Fe uptake and translocation (Zhai et al., 2017).

Among the signaling networks that orchestrate these responses, the mitogen-activated protein kinase (MAPK) cascade functions as a pivotal pathway enabling plants to perceive and transduce external stress cues (Yadav et al., 2021). MAPK modules typically operate through sequential phosphorylation (MAPKKK → MAPKK → MAPK) to regulate downstream transcription factors, thereby initiating defense mechanisms against metal toxicity (Takatsuji, 1998). In Arabidopsis, the *MEKK1*–*MKK4*/*MKK5*–*MPK3*/*MPK6* cascade confers resistance to both abiotic and biotic stresses (Xu et al., 2019). Similarly, in rice, infection by *Magnaporthe oryzae* triggers endoplasmic-reticulum-stress-mediated activation of MAPK cascades to enhance disease resistance (Li et al., 2021). In potato (*Solanum tuberosum*), the *StMEK1*-mediated MAPK cascade plays a pivotal role in immunity against pathogens (Khan et al., 2021) whereas in cotton (*Gossypium hirsutum*) the *GhMAP3K65* gene modulates pathogen perception via salicylic acid, jasmonic acid, and ethylene signaling, as well as ROS homeostasis; silencing *GhMAP3K65* significantly enhances resistance to *Ralstonia solanacearum* (Ammar et al., 2025).

Beyond biotic stresses, MAPK pathways are also integral to heavy-metal tolerance. Under Zn^2+^ stress, MYB72 protein orchestrates Zn^2+^ uptake and detoxification (Peralta et al., 2022); Cd^2+^ exposure activates *MPK3* and *MPK6* via ROS accumulation to enhance Cd^2+^ tolerance in Arabidopsis (Liu et al., 2010); Cr^3+^ stress induces the bZIP transcription factor TGA3 to promote H_2_S biosynthesis through transcriptional activation of LCD, thereby improving Cr^3+^ resistance (Fang et al., 2017); Pb^2+^ exposure up-regulates *RsWRKY* to modulate the antioxidant system (Khedia et al., 2019); Ni^2+^ stress is mitigated by *SbMYB15* regulated antioxidant enzyme activities (Sapara et al., 2019); and As tolerance is conferred by OsARM1-mediated control of As uptake and root-to-shoot translocation (Wang et al., 2017). Collectively, these studies demonstrate that MAPK cascades initiate heavy-metal stress defense by phosphorylation-dependent activation of downstream transcription factors.

Consistent cross-species multi-omics evidence has established the MAPK cascade as the convergent node that integrates ROS, NO, and hormonal signals during heavy-metal stress, directly phosphorylating key transcription factors such as WRKY, MYB, and bZIP to activate antioxidant and ion-homeostatic networks (Li et al., 2022). Nevertheless, the expression patterns of the MAPK module and its Cu^2+^-specific defense logic in peanut under Cu^2+^ stress remain undocumented.

To address this knowledge gap, we exposed peanut seedlings to 0 and 50 mg/L Cu^2+^ for 48 hours using a hydroponic system. By combining root transcriptomics with physiological readouts, we aim to elucidate the response mechanisms of the MAPK signaling pathway, antioxidant enzymes, and transcription factor DEGs in peanut roots to copper stress, thereby providing a theoretical basis for breeding heavy-metal-resistant crops.

## Materials and methods

### Material cultivation

Seeds of peanut cultivar ‘*Haihua No. 1*’ were used. Intact, uniformly sized seeds were selected on the basis of 100-seed weight and kernel diameter using a caliper and analytical balance. Selected seeds were surface-sterilized with 70% (v/v) ethanol for 10 min, rinsed three times with sterile deionized water, and germinated on moist filter paper in Petri dishes at 28 ± 1 °C in darkness. Distilled water was replenished every 12 h to maintain constant moisture. After 72 h, the seedlings were cultured in distilled water for 4 days at 25 °C to be used as experimental material.

### Experimental design

The experiment consisted of two treatment groups: a distilled water control (CK) and a copper stress treatment (Cu). The copper stress treatment was administered using 50 mg/L of CuSO_4_·H_2_O. Each treatment was applied for 48 hours with three biological replicates.

After the treatment, we photographed the samples to document their growth status. Subsequently, we harvested the roots. A portion of the root samples was immediately frozen in liquid nitrogen and stored at -80 °C for subsequent physiological, transcriptome (RNA-seq), and RT-qPCR analyses. The remaining samples were blanched at 90 °C for 30 minutes, then oven-dried to a constant weight at 60 °C to measure the content of various ions in the tissues.

### Metrics and analysis methods

#### Growth phenotype of peanut seedling roots

The growth status of the peanut seedlings was documented by taking high-resolution photos with a smartphone. Subsequently, the roots were harvested, and root length was measured with a caliper, while fresh root weight was determined using an electronic balance.

#### Analysis of oxidative stress indicators and antioxidant enzyme activities in peanut seedling roots

Frozen peanut root samples stored at -80 °C were used to determine several stress-related physiological indices. These included the content of reactive oxygen species (ROS), specifically superoxide anion (O_2_^-^) and hydrogen peroxide (H_2_O_2_), as well as malondialdehyde (MDA). We also measured the activities of three antioxidant enzymes: superoxide dismutase (SOD), catalase (CAT), and peroxidase (POD) (Dong et al., 2023).

#### Absolute quantification of eight cations in peanut seedling roots

Dried samples were digested in 5 mL HNO_3_/HClO_4_ solution (5:1, v/v) at 180 °C until the solution became clear. The resulting digest was then brought to a final volume of 25 mL with ultrapure water. The absolute content of the eight cations (K^+^, Na^+^, Ca^2+^, Mg^2+^, Fe^3+^, Mn^2+^, Cu^2+^, and Zn^2+^) was determined using flame atomic absorption spectrophotometry (AAS, PinAAcle 900T, PerkinElmer, USA). The measurements were validated using matrix-matched standards and a certified reference material (NIST 1573a).

#### High-throughput transcriptome sequencing (RNA-seq)

Total RNA was extracted from peanut roots stored at −80 °C using TRIzol reagent (Invitrogen, Carlsbad, CA, USA) according to the manufacturer’s protocol. RNA integrity was assessed using an Agilent Bioanalyzer 2100 system with the RNA Nano 6000 LabChip kit (Agilent Technologies, Santa Clara, CA, USA), and only samples with an RNA integrity number (RIN) ≥ 8.0 were used for subsequent steps.

Strand-specific cDNA libraries were constructed from 2 µg of total RNA using the TruSeq RNA Sample Preparation Kit v2 (Illumina, San Diego, CA, USA) according to the manufacturer’s guidelines. Briefly, poly(A)-enriched mRNA was purified with oligo(dT)-conjugated magnetic beads, fragmented, and reverse-transcribed into cDNA. The cDNA was then end-repaired, A-tailed, and ligated to indexed adapters. After 12 cycles of PCR amplification (98 °C for 30 s; 12 cycles of 98 °C for 10 s, 60 °C for 30 s, 72 °C for 30 s; 72 °C for 5 min), the libraries were quantified with a Qubit 3.0 fluorometer (Thermo Fisher Scientific) and validated on the Bioanalyzer 2100. Paired-end 150-bp sequencing was performed on the Illumina HiSeq 4000 platform by LC Sciences (Hangzhou, China), generating approximately 6 Gb of clean data per sample (Dong et al., 2023). Our raw sequencing data have been uploaded to the NCBI database (https://www.ncbi.nlm.nih.gov/). The accession ID for the Sequence Read Archive (SRA) dataset is SUB15570566.

#### Quantitative real-time polymerase chain reaction assay

Total RNA was extracted from root samples, which had been snap-frozen in liquid nitrogen and stored at −80 °C to prevent RNA degradation. The extraction was performed using the RNAiso Plus reagent (Takara Bio, Kusatsu, Japan) according to the manufacturer’s instructions. Subsequently, mRNA was enriched from the total RNA using oligo(dT)-conjugated magnetic beads (Invitrogen, Carlsbad, CA, USA) and further purified via probe-based hybridization. First-strand cDNA was synthesized with the GoScript™ Reverse Transcription System (Promega, Beijing, China) from 1 µg of purified mRNA.

Quantitative real-time PCR was performed on a QuantStudio™ 5 Real-Time PCR System (Thermo Fisher Scientific, Waltham, MA, USA) using GoTaq^®^ qPCR Master Mix (Promega, Beijing, China) in a 20 µL reaction volume containing 2 µL of 1:10-diluted cDNA, 0.4 µM each primer, and 1 × master mix. Cycling conditions were 95 °C for 3 min, followed by 40 cycles of 95 °C for 15 s and 60 °C for 30 s. Melting-curve analysis (65–95 °C, 0.5 °C increment) confirmed amplicon specificity.

Actin-7 (GenBank ID: *LOC112715878*) served as the internal reference gene. Relative mRNA abundance was calculated by the 2^(−ΔΔCt)^ method (Sun et al., 2024). Primer sequences and amplicon sizes are listed in **Supplementary Table S1**.

### Data processing and statistical analysis

Raw data for root growth parameters, physiological indices, and RT-qPCR were organized and normalized in Microsoft Excel 2016.

After quality filtering with Trimmomatic v0.39, clean reads were aligned to the *Arachis hypogaea* reference genome (NCBI Taxonomy ID 3818) using HISAT2 v2.2.1 (Kim et al., 2019) Gene-level counts were generated with featureCounts v2.0.3 (Liao et al., 2014) Differentially expressed genes (DEGs) were identified using edgeR v3.38.0 (Robinson et al., 2010) under thresholds of |log_2_ fold change| ≥ 1 and FDR < 0.05.

GO and KEGG enrichment of DEGs and was conducted using clusterProfiler v4.0 with a hypergeometric test (*p* < 0.05, Benjamini–Hochberg correction).

For physiological phenotypes, ion contents, and RT-qPCR data, one-way analysis of variance (ANOVA) was performed in R v4.3.1 using the aov() function, followed by Duncan’s multiple range test (α = 0.05).

Data visualization was performed as follows: bar plots of physiological data and KEGG enrichment results were generated in Origin 2021; heatmaps were constructed with TBtools-II (Chen et al., 2023).

## Results

### Root growth, oxidative stress, and antioxidant responses in peanut seeding roots under copper stress

**Figure 1:**
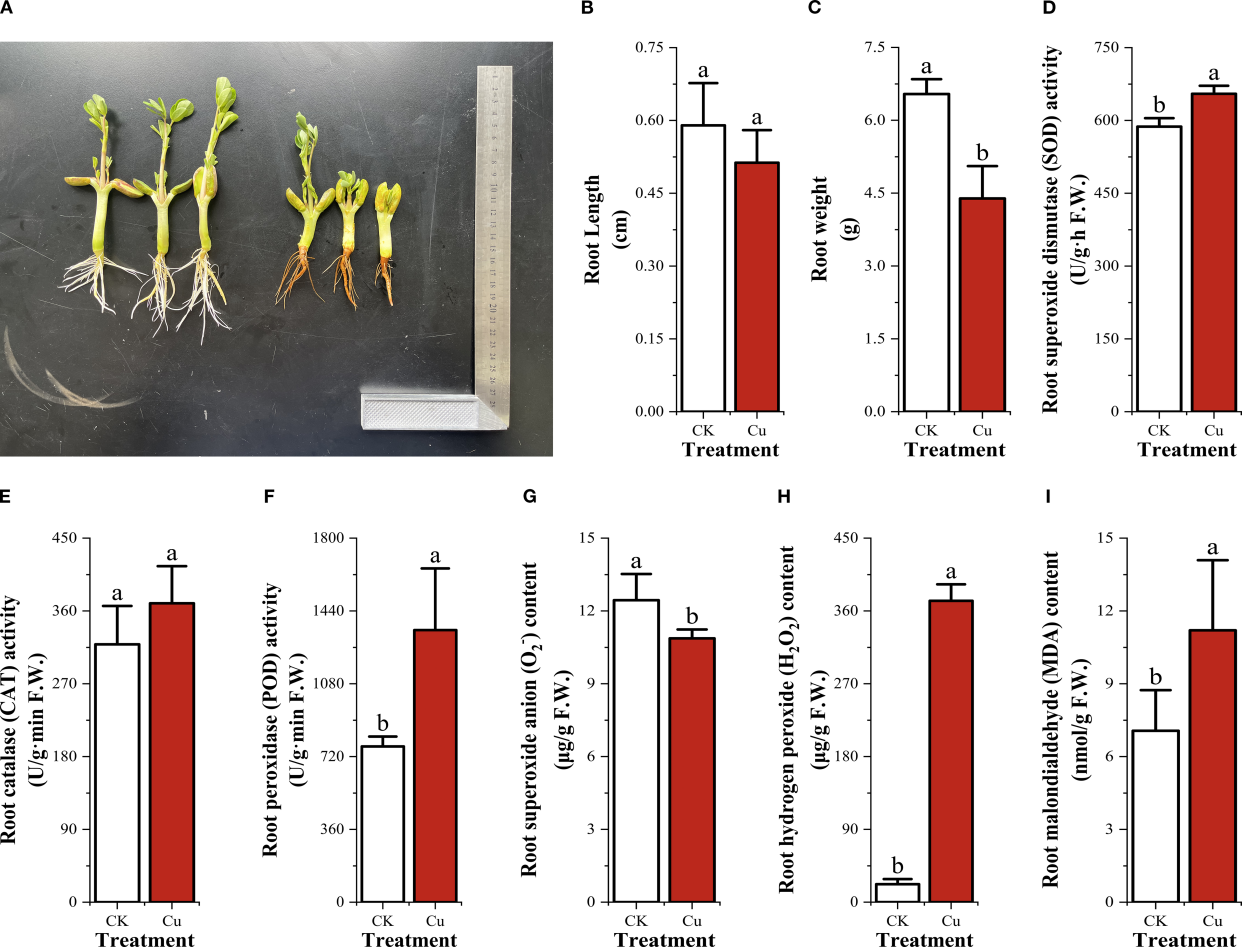
Root growth, secondary oxidative stress and antioxidant enzyme activities in peanut seedling roots under two treatments. **(A)** Root growth photographs; **(B)** Root length; **(C)** Root weight; **(D)** Superoxide dismutase (SOD) activity; **(E)** Catalase (CAT) activity; **(F)** Peroxidase (POD) activity; **(G)** Superoxide anion (O_2_^-^) content; **(H)** Hydrogen peroxide (H_2_O_2_) content; **(I)** Malondialdehyde (MDA) content.

Excess Cu^2+^ markedly inhibited root elongation and biomass accumulation in peanut seedlings. Compared with the control (CK), 50 mg/L Cu^2+^ treatment reduced mean primary root length by 32.9% (CK: 6.540 cm vs. Cu^2+^: 4.390 cm) and fresh root weight by 18.6% (CK: 0.590 g vs. Cu^2+^: 0.480 g). Concurrently, Cu^2+^ stress significantly elevated antioxidant enzyme activities (**Figures 1A–C**). Superoxide dismutase (SOD) activity increased from 587.077 U g^-^¹ h^-^¹ in CK to 654.867 U g^-^¹ h^-^¹ under Cu^2+^ stress; catalase (CAT) activity rose from 318.723 to 369.33 U g^-^¹ min^-^¹, while peroxidase (POD) activity exhibited the most pronounced induction, increasing 1.75-fold (from 769.807 to 1 345. 14 U g^-^¹ min^-^¹). The oxidative burst profile revealed that superoxide anion (O_2_^-^) content declined slightly (CK: 12.44 μg/g; Cu^2+^: 10.873 μg/g), whereas hydrogen peroxide (H_2_O_2_) and malondialdehyde (MDA) accumulated markedly. H_2_O_2_ content increased >16-fold (CK: 22.08 μg/g; Cu^2+^: 372.533 μg/g), and MDA concentration rose 1.59-fold (CK: 7.06 μmol/g; Cu^2+^: 11.2 μmol/g) (**Figures 1D–F**). These data demonstrate that excess Cu^2+^ elicits secondary oxidative stress in peanut roots, triggering a compensatory up-regulation of the enzymatic antioxidant system (SOD, CAT, and POD) to mitigate Cu^2+^-induced ROS accumulation and membrane lipid peroxidation.

### Eight metal ion concentrations in peanut seeding roots under copper stress

**Figure 2:**
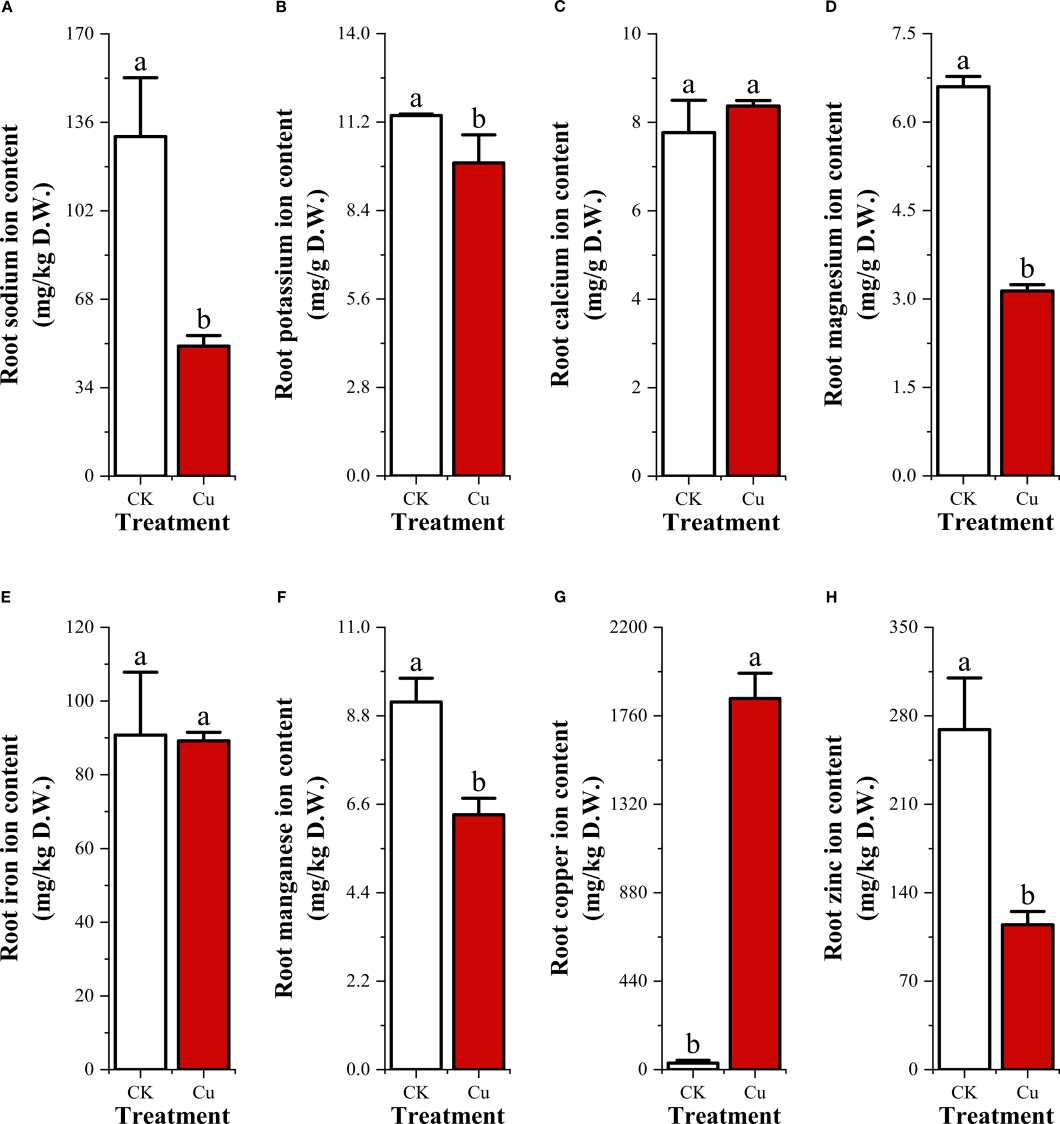
Contents of 8 metal cations in peanut seedling roots under two treatments. **(A)** Sodium ion (Na^+^); **(B)** Potassium ion (K^+^); **(C)** Calcium ion (Ca^2+^); **(D)** Magnesium ion (Mg^2+^); **(E)** Zinc ion (Zn^2+^); **(F)** Manganese ion (Mn^2+^); **(G)** Iron ion (Fe^3+^); **(H)** Copper ion (Cu^2+^).

After 48 h of exposure, ionomic profiling revealed marked shifts in root metal.

contents (**Figure 2**). Sodium (Na^+^) exhibited the sharpest decline, dropping by 61.6%from 130.517 mg/kg in the control (CK) to 50.033 mg/kg under Cu^2+^ stress. Potassium (K^+^) and calcium (Ca^2+^) showed modest variations: K^+^ decreased from 11.407 to 9.577mg/g (-16.0%), whereas Ca²^+^ slightly increased from 7.77 to 8.557 mg/g (+10.1%), although neither change reached statistical significance (*p < 0.05*).

Among divalent micronutrients, magnesium (Mg^2+^) fell by 52.5% (CK:6.6 mg/g; Cu^2+^:3.137 mg/g), zinc (Zn^2+^) by 57.4% (269.1 vs 114.547 mg/kg), and manganese (Mn^2+^) by 30.7% (9.143 vs 6.34 mg/kg). Iron (Fe^3+^) displayed only a marginal reduction of 1.8% (90.793 vs 89.173 mg/kg).

In contrast, copper (Cu^2+^) accumulation was dramatic. Root Cu^2+^ concentration surged 57.9-fold from 31.867mg/kg in CK to 1846.603 mg/kg under Cu^2+^ stress.

Collectively, the data indicate that excess exogenous Cu²^+^ not only caused extreme Cu^2+^ accumulation but also triggered substantial losses of Na^+^, K^+^, Mg^2+^, Zn^2+^, and Mn^2+^, while Ca^2+^ and Fe^3+^ remained relatively stable. This ionic imbalance underscores the severity of Cu^2+^-induced nutrient deficiency stress in peanut roots.

### DEGs and enrichment analysis of peanut seedling roots under copper stress

**Figure 3:**
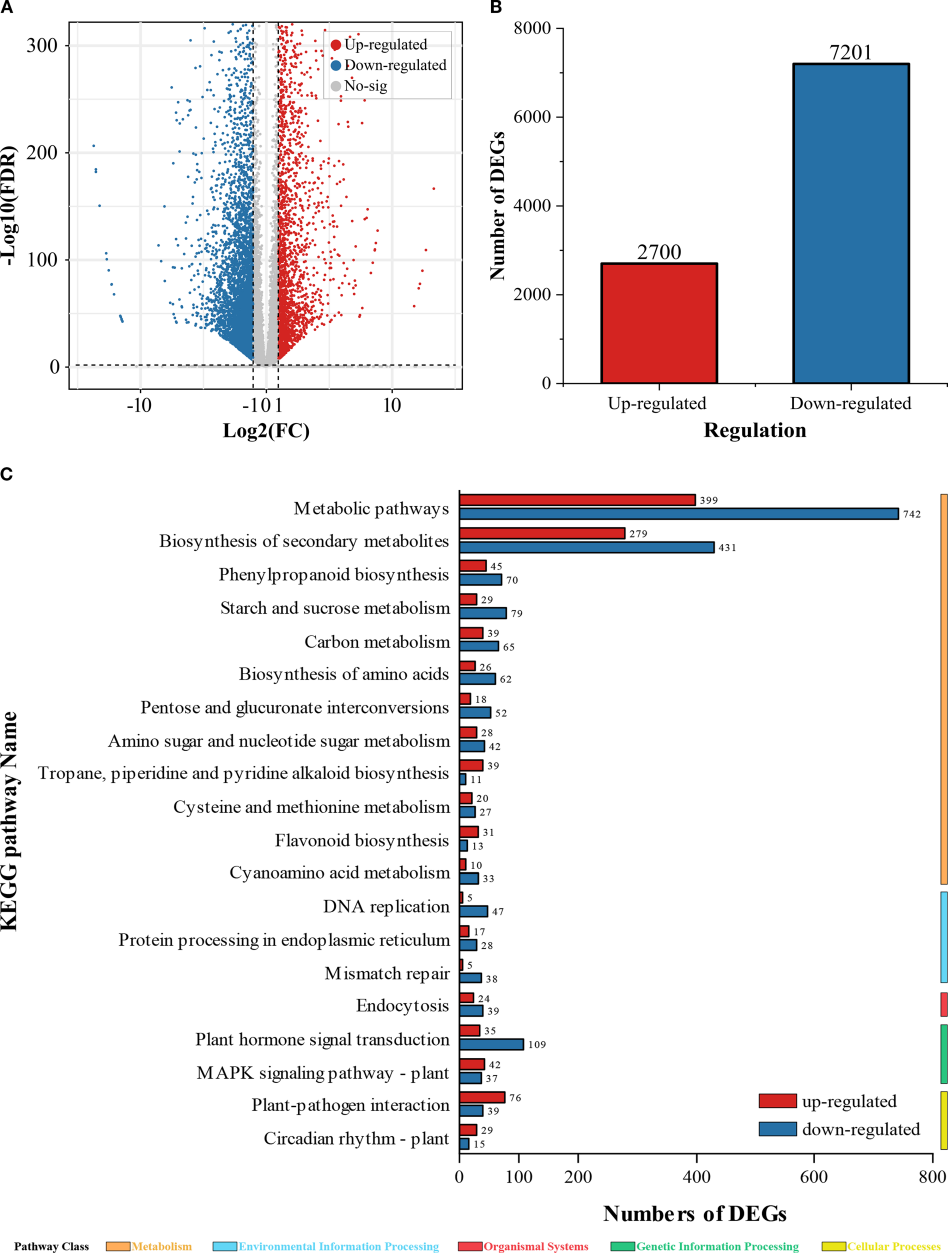
DEGs and KEGG enrichment analysis. **(A)** Volcano plot of DEGs; **(B)** Total count of up-regulated and down-regulated DEGs; **(C)** KEGG enrichment analysis of DEGs.

Following 48 h of Cu^2+^ exposure, volcano plots revealed a striking transcriptional reprogramming (**Figure 3**). A total of 9901 DEGs were identified, with 2700 genes up-regulated and 7201 genes down-regulated, indicating that Cu^2+^ stress exerts a predominantly repressive effect on root gene expression.

KEGG pathway enrichment analysis further uncovered significant perturbations in primary and secondary metabolism. Within the global “metabolic pathways” (ko01100), 743 genes were suppressed while only 399 were induced (down/up ratio = 1.86). This pronounced skew suggests a broad inhibition of carbohydrate, amino-acid, and energy metabolism, likely compromising ATP-dependent ion uptake and exacerbating nutrient deficiency.

Similarly, the “biosynthesis of secondary metabolites” (ko01110) displayed 431 down-regulated versus 279 up-regulated genes (down/up ratio =1.54). The disproportionate suppression of this pathway implies that Cu^2+^ stress hampers the production of flavonoids, phenylpropanoids, and other stress-protective compounds thereby weakening the root’s antioxidant defense network.

These transcriptomic signatures reveal that excess Cu^2+^ not only triggers massive transcriptional repression but also targets metabolic and secondary-metabolite pathways that are central to energy homeostasis and oxidative stress mitigation in peanut roots.

**Figure 4:**
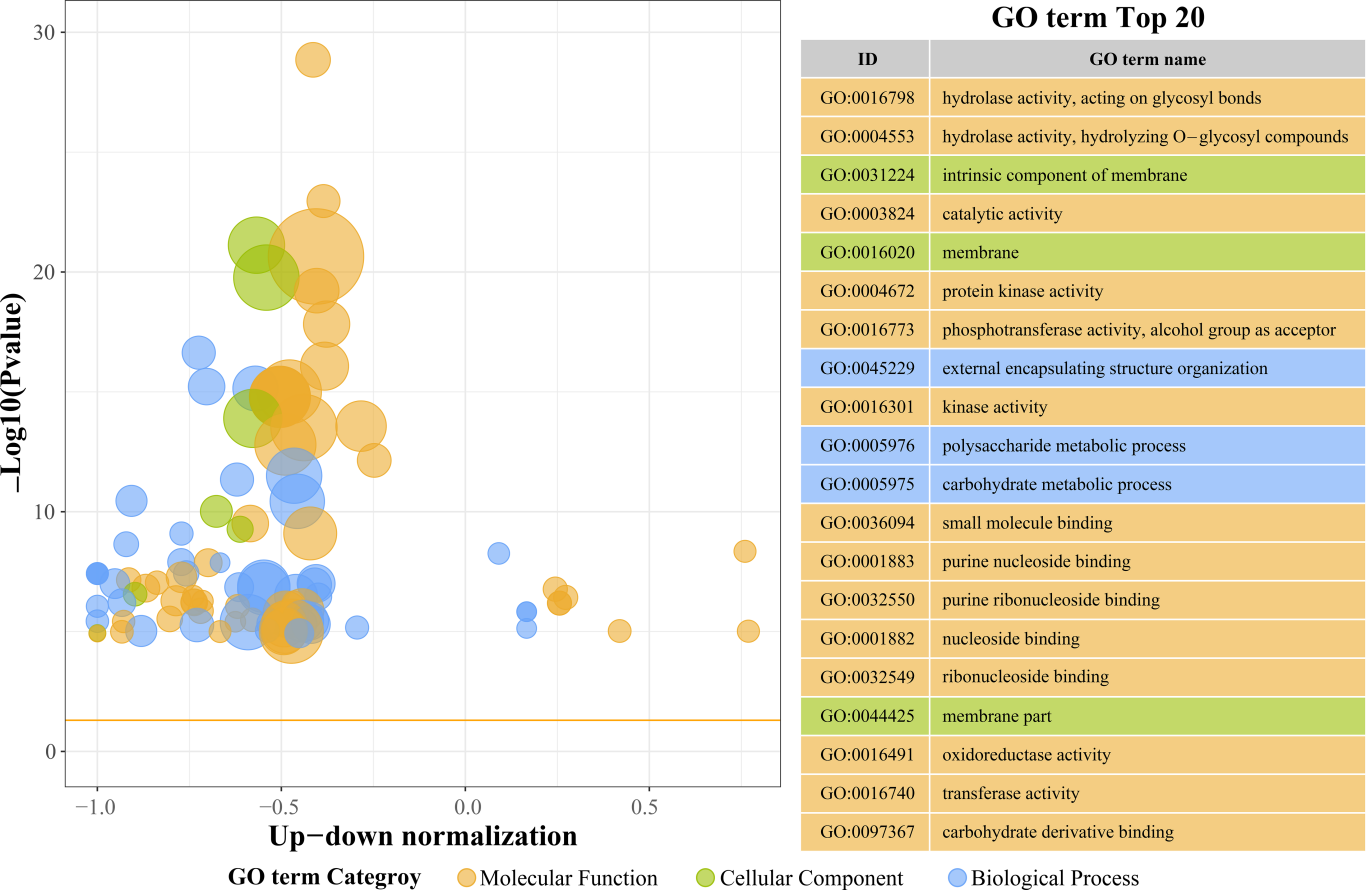
The top 20 GO terms with the smallest p-values in the Gene Ontology (GO) enrichment enrichment analysis.

**Figure 4** summarizes the Gene Ontology (GO) enrichment of differentially expressed genes (DEGs) in peanut roots exposed to 48 h of Cu^2+^ stress, focusing on the 20 terms with the smallest *p*-values. These categories illuminate the principal biological responses triggered by excess Cu^2+^.

From a Molecular Function (MF) perspective, we found the terms for hydrolase activity acting on glycosidic bonds (GO:0016798), hydrolase activity, hydrolyzing O-glycosyl compounds (GO:0004553), and general catalytic activity (GO:0003824). This means that hydrolase, oxidoreductase, and transferase pathways were significantly activated during Cu²^+^ stress. It is worth noting that oxidoreductase activity (GO:0016491) and transferase activity (GO:0016740) showed relatively weaker enrichment, which suggests that plants may primarily utilize glycosyltransferases and related enzymes to synthesize secondary metabolites to promote Cu²^+^ chelation and detoxification. Meanwhile, terms such as intrinsic component of membrane (GO:0031224) and plasma membrane (GO:0016020) were significantly enriched, which emphasizes the critical role of membrane-associated structures in Cu²^+^ perception and stress signaling. The extracellular region (GO:0044425) also showed a modest enrichment, indicating that membrane-resident peroxidative repair systems contribute to cell wall integrity under Cu²^+^ stress. Additionally, in terms of Biological Process (BP) terms, external encapsulating structure organization (GO:0045229) was the most prominent process, suggesting that plants rapidly strengthen their cell wall architecture to form a physical barrier, thereby limiting Cu²^+^ influx into root tissues. Polysaccharide metabolic process (GO:0005976) and carbohydrate metabolic process (GO:0005975) showed weaker enrichment, which indicates that short-term (48 h) Cu²^+^ stress prioritizes immediate defense responses, meaning that plant carbohydrate metabolism is affected.

### Antioxidant-related DEGs in peanut seedling roots under copper stress

**Figure 5:**
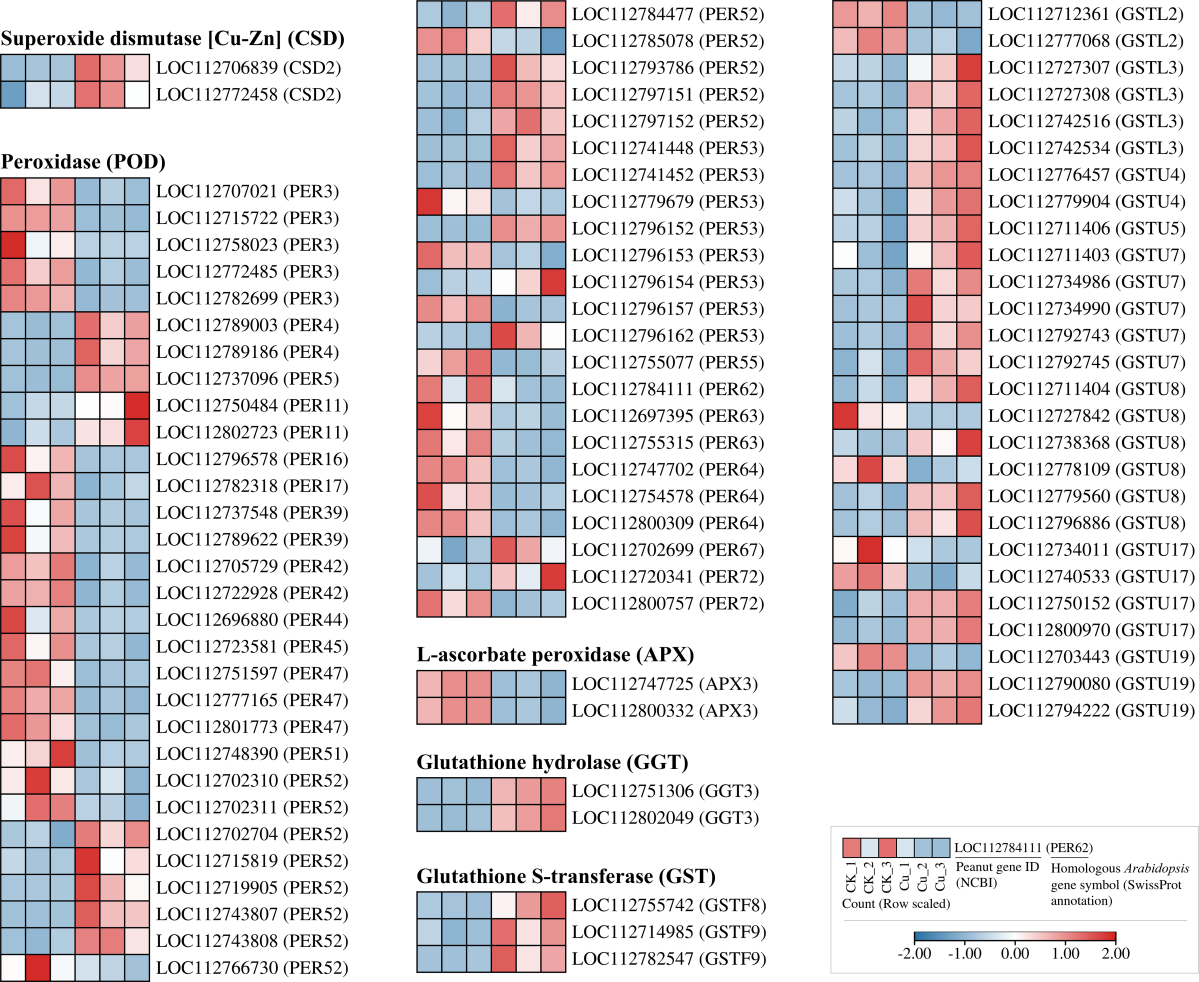
Heatmap of differentially expressed genes encoding antioxidant-related enzymes in peanut seedling roots under two treatments. CSD, Copper/Zinc Superoxide Dismutase; PER, Peroxidase; APX, Ascorbate Peroxidase; GGT, Gamma-Glutamyltransferase; GST, Glutathione S-Transferase; GSTU, Glutathione S-Transferase U.

Some DEGs related to antioxidant enzyme coding and glutathione metabolism were listed in **Figure 5**. We found that under copper stress, only two genes encoding copper/zinc superoxide dismutase (Cu/Zn-SOD, CSD), *LOC112706839* and *LOC112772458*, were transcriptionally upregulated. Interestingly, no DEGs encoding catalase (CAT) were detected. In stark contrast, the peroxidase (POD) superfamily exhibited a significant transcriptional response. A total of 53 DEGs encoding PER enzymes were identified, distributed across 20 subtypes, encoding PER3, PER4, PER52, and PER53. Notably, the PER52 subtype alone comprised 13 DEGs, suggesting a recent gene duplication event that may have conferred a selective advantage in reactive oxygen species (ROS) scavenging during Cu^2+^ stress. Additionally, two DEGs encoding L-ascorbate peroxidase (APX), *LOC112747725* and *LOC112800332*, were also upregulated. This indicates that the transcriptional activation of POD enzyme-encoding genes plays a dominant role in the secondary oxidative stress response of peanut roots to copper stress.

The glutathione S-transferase (GST) superfamily contained 31 members, categorized into three subfamilies: GSTF (3 DEGs), GSTL (6 DEGs), and GSTU (21 DEGs). Almost all GST genes were upregulated, with the GSTU class showing the strongest response. Concurrently, two DEGs encoding glutathione hydrolase (GGT), *LOC112751306* and *LOC112802049*, were also upregulated by copper stress. Collectively, these findings reveal that the conversion and hydrolysis of glutathione are crucial mechanisms for peanut roots to cope with copper stress, acting synergistically with the antioxidant enzyme system.

### DEGs of mitogen-activated protein kinase cascade in peanut seedling roots under copper stress

**Figure 6:**
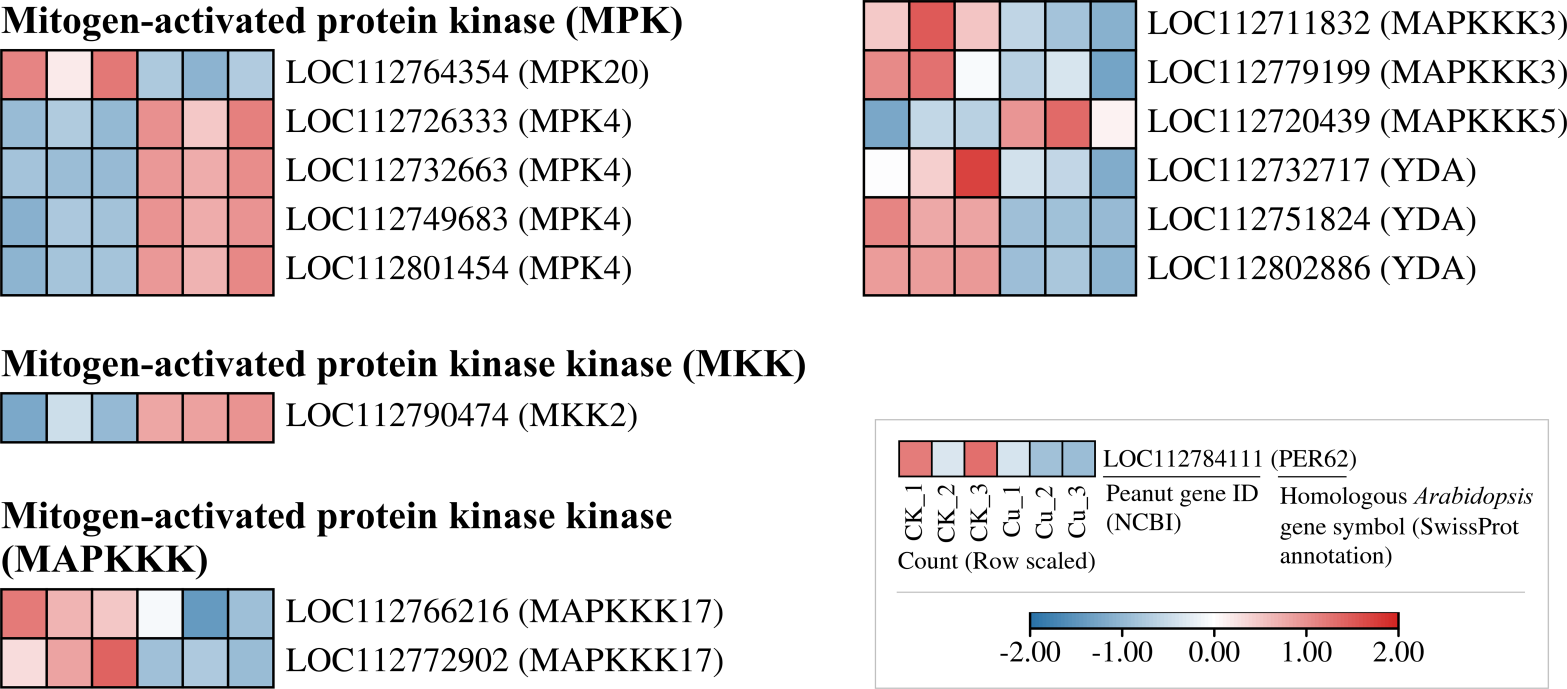
Heatmap of differentially expressed genes encoding MAPK signaling-related proteins in peanut seedling roots under two treatments. MPK, Mitogen-Activated Protein Kinase; MKK, Mitogen-Activated Protein Kinase Kinase; MAPKKK, Mitogen-Activated Protein Kinase Kinase Kinase; YDA, MAPKKK YODA.

**Figure 6** listed some of the copper-responsive DEGs encoding MAPK signaling pathway proteins. We found that all five DEGs encoding MPK were upregulated by copper stress, and the upstream gene encoding MKK2, LOC112790474, was also upregulated. In contrast, most of the DEGs encoding MAPKKK isoforms were transcriptionally suppressed by Cu^2+^. Despite the transcriptional suppression of MAPKKK at the sampling time point, it is interesting to note that the transcription of its downstream MKK and MAPK modules was activated by copper stress. This finding may suggest that the core downstream modules of the MAPK signaling pathway are activated in response to copper stress, thereby playing a role in coping with the stress.

### Transcription Factor coding DEGs in peanut seedling roots under copper stress

**Figure 7:**
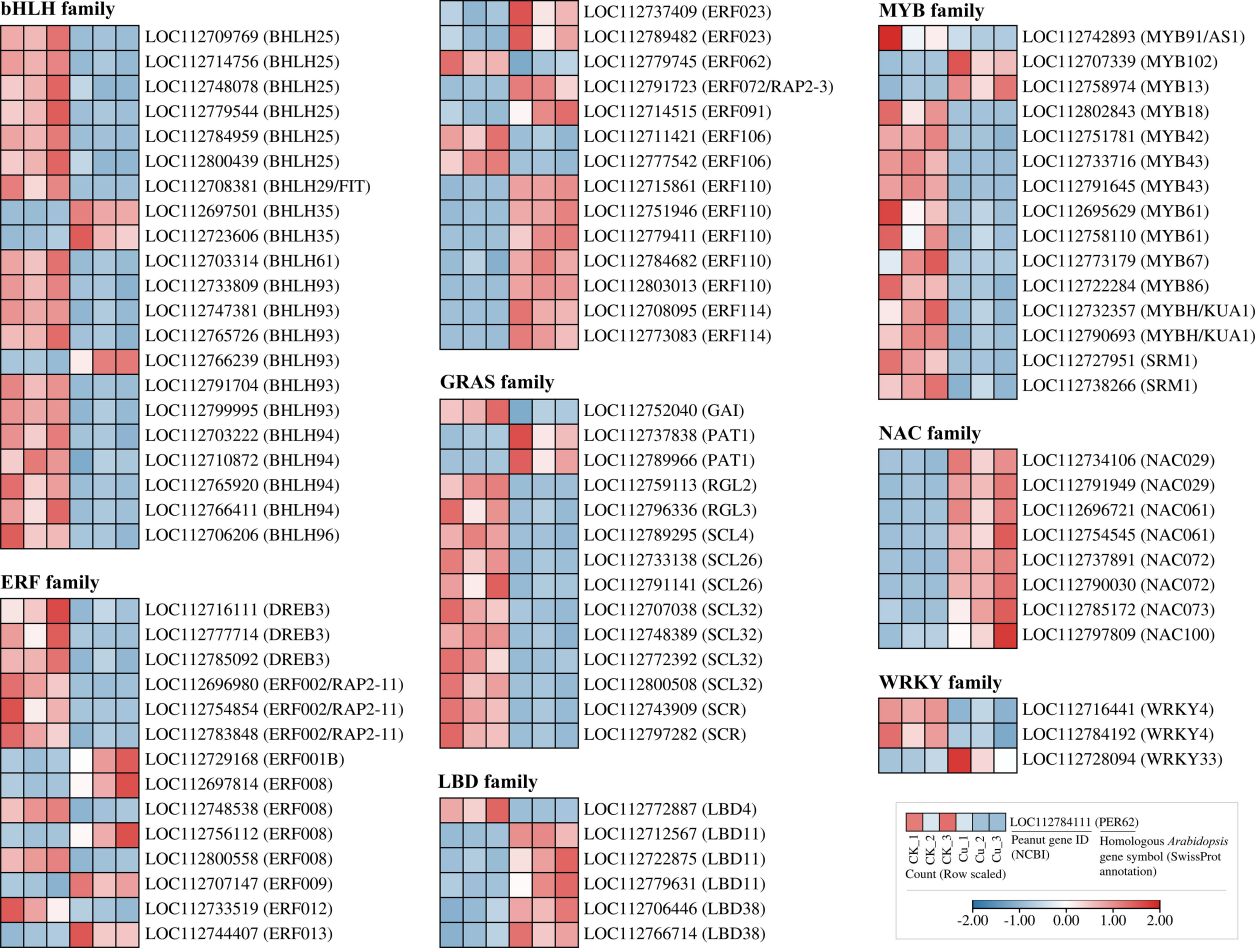
Heatmap of differentially expressed genes encoding transcription factors from seven families in peanut seedling roots under two treatments. bHLH, basic Helix-Loop-Helix; ERF, Ethylene Response Factor; DREB, Dehydration-Responsive Element-Binding Protein; RAP2, Related to AP2; GRAS, GRAS; GAI, GIBBERELLIN INSENSITIVE; PAT, PAT; RGL, RGA-LIKE; SCL, SCARECROW-LIKE; SCR, SCARECROW; LBD, Lateral Organ Boundaries Domain; MYB, Myeloblastosis; KUA, Kuan; SRM, Sorem; NAC, NAC domain protein; WRKY, WRKY domain protein.

We also investigated the response of transcription factor-encoding DEGs to copper stress, and in **Figure 7**, we found a large number of DEGs in eight common transcription factor (TF) families. Notably, some families showed a significant downregulation trend: 19 out of 21 DEGs in the bHLH family were consistently downregulated under copper stress. A similar trend was observed in the GRAS family (13 out of 15 DEGs) and the MYB family (13 out of 15 DEGs). In stark contrast, all DEGs from the NAC family (8 DEGs) and the LBD family (5 DEGs) were strongly upregulated by copper stress. This suggests that while most transcription factor genes are suppressed, members of the NAC and LBD families may be specifically responsible for regulating downstream stress and adaptive pathways in response to Cu^2+^ toxicity.

### Quantitative real-time polymerase chain reaction validation of high-throughput transcriptome sequencing (RNA-seq)

To validate the accuracy of the RNA sequencing (RNA-seq) data, we randomly selected 18 DEGs for quantitative real-time PCR (RT-qPCR) validation. These 18 genes included 4 DEGs related to the MAPK signaling pathway, 8 encoding transcription factor proteins, 2 encoding peroxidase (POD) enzymes, 2 encoding glutathione S-transferase, and 2 additional randomly selected genes.

**Figure 8:**
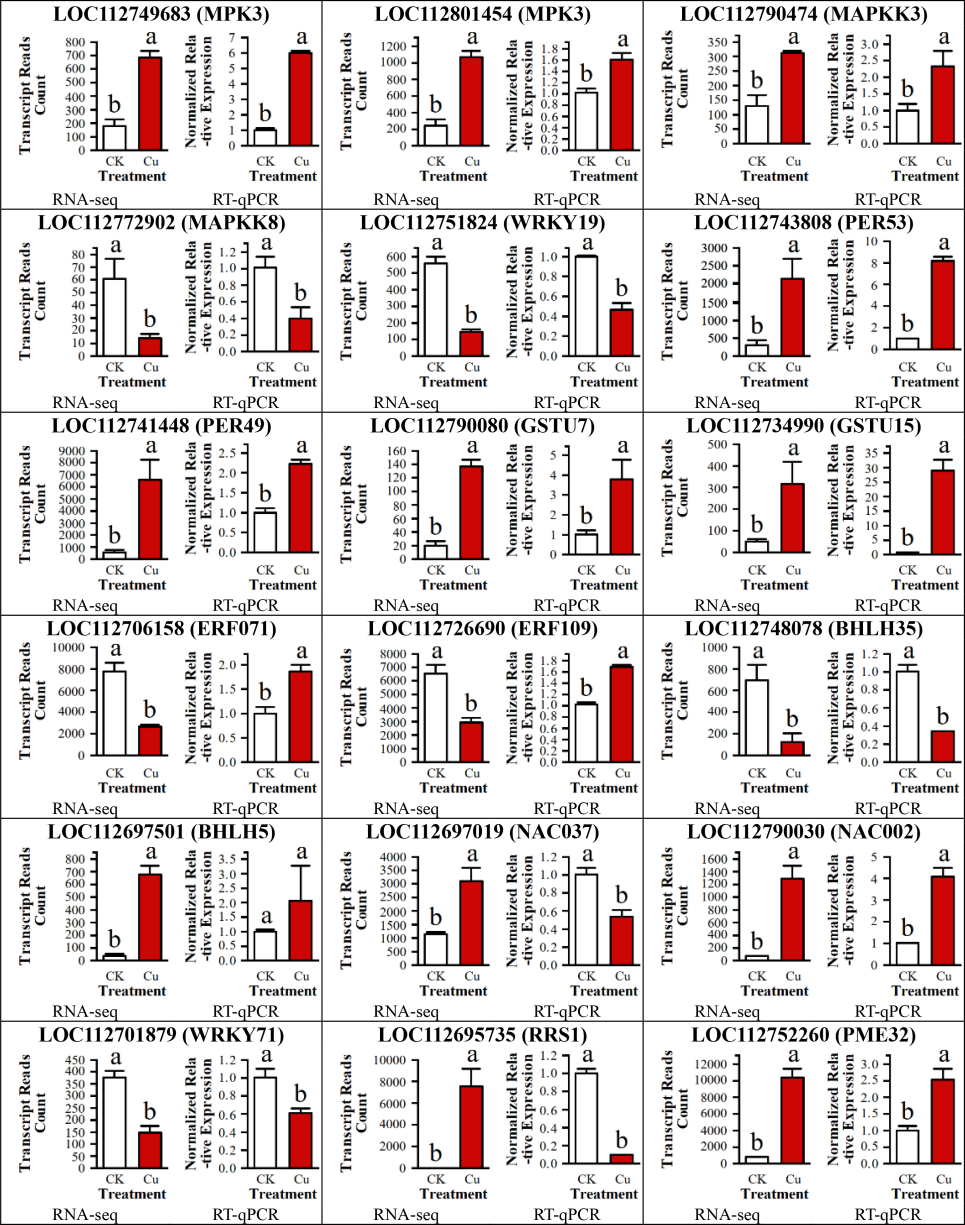
RT-qPCR validation of 18 DEGs in peanut seedling roots under two treatments. MAPKK, Mitogen-Activated Protein Kinase Kinase; WRKY, WRKY Transcription Factor; PER, Peroxidase; GSTU, Glutathione S-Transferase U; ERF, Ethylene Response Factor; bHLH, basic Helix-Loop-Helix; NAC, NAC Transcription Factor; RRS, Resistance to Root Rot Symptom; PME, Pectin Methylesterase.

As shown in **Figure 8**, among the 18 DEGs, the expression trends of 14 genes were consistent between the two testing methods, indicating a high reliability of the RNA-seq data. However, the RT-qPCR results for four DEGs (*LOC112706158* (*ERF071*), *LOC112726690* (*ERF109*), *LOC112697019* (*NAC037*) and *LOC112695735* (*RRS1*)) were inconsistent with the RNA-seq results, which might be attributed to technical differences between the methods or the dynamic nature of gene expression.

## Discussion

### Secondary oxidative stress and antioxidation in peanut roots under copper stress

Elevated Cu^2+^ concentrations not only disrupt normal cellular physiology but also inhibit both cell division and elongation, ultimately retarding overall plant growth. In the present study, Cu^2+^ exposure induced pronounced oxidative stress in peanut roots, as evidenced by a 1.5-fold increase in malondialdehyde (MDA) content relative to the control. This elevation indicates extensive peroxidation of membrane lipids and, consequently, severe oxidative injury to root cell membranes (Saleem et al., 2020).

Interestingly, although superoxide anion (O_2_^-^) levels decreased, hydrogen peroxide (H_2_O_2_) accumulated 17-fold under Cu^2+^ stress. This apparent paradox aligns with the dual role of H_2_O_2_ as both a reactive oxygen species and a signaling molecule. At elevated concentrations, H_2_O_2_ operates in a dose-dependent manner to activate antioxidant defenses and to modulate the expression of stress-responsive genes, thereby exerting profound regulatory effects on plant growth and development (Khedia et al., 2019).

Under copper stress, the activities of three key antioxidant enzymes in peanut seedling roots increased markedly: superoxide dismutase (SOD) rose by 11%, catalase (CAT) by 16%, and peroxidase (POD) by 75% (**Figure 1**). This concerted up-regulation indicates that peanut initiates a robust enzymatic antioxidant defense to mitigate Cu^2+^-induced reactive oxygen species (ROS) accumulation and to maintain cellular redox homeostasis.

This pattern aligns with previous reports in which cadmium or other heavy-metal stresses similarly enhance SOD and CAT activities to quench ROS and limit oxidative damage (Liu et al., 2014). The disproportionately strong induction of POD observed here, however, suggests that peanut may deploy a distinct POD-centric detoxification strategy under Cu^2+^ stress. Whether this reflects an isoform-specific POD expansion, substrate preference, or interaction with Cu^2+^-chelating metabolites remains to be elucidated.

### Oxygen ion content in peanut roots under copper stress

Copper stress elicited pronounced perturbations in the concentrations of eight metal ions in peanut roots (**Figure 2**). As we observed, Cu^2^+^^ exhibited a dramatic surge (≈ 58-fold), while the levels of Na^+^, K^+^, Mg^2+^, Zn^2+^, and Mn^2+^ declined significantly.

Integrated cation contents data and RNA-seq evidence suggests that this disruption of ion balance is mechanistically linked to the transcriptional regulation of cation-transport genes. The underlying mechanism involves competitive binding to shared transporters, such as cation channels, heavy-metal ATPases (HMAs), and natural resistance-associated macrophage proteins (NRAMPs). Specifically, the downregulation of shaker-type K^+^ channel transcripts (e.g., AKT/KAT homologs) and high-affinity K^+^ transporters (HKTs) directly reduces K^+^ uptake, while the concomitant suppression of ZIP and NRAMP family members restricts the acquisition of Zn^2+^ and Mn^2+^ (Gao et al., 2008; Kazemi-Dinan et al., 2014; Xu et al., 2015).

Concurrently, the plant initiates active defense mechanisms. We observed the upregulation of genes encoding Cu^2+^-chelating agents (e.g., metallothioneins, phytochelatin synthases) and Cu^2+^-transporting P-type ATPases (HMAs), which facilitates the intracellular sequestration of Cu^2+^ and its vacuolar compartmentalization. This process further exacerbates Cu^2+^ enrichment in the roots while simultaneously intensifying the deficiencies of competing cations due to impaired uptake. Furthermore, Cu^2+^-induced oxidative stress compromises membrane integrity and ion selectivity, and the inhibition of root elongation along with the reduction in root-hair density indirectly exacerbates these nutrient deficiencies.

Nevertheless, the marked decline in Zn^2+^ levels is likely to exert deleterious effects on plant growth and development, as zinc functions as an essential catalytic component of numerous enzymes and is indispensable for photosynthesis, respiration, and other vital physiological processes (Kazemi-Dinan et al., 2014). Collectively, these findings demonstrate that copper stress imposes severe negative impacts on peanut growth and, under extreme conditions, may accelerate the onset of senescence and death in peanut as well as in other plant species.

### The effect of copper stress on gene transcription in peanut roots

RNA-seq analysis revealed extensive transcriptional reprogramming in peanut seedling roots under Cu^2+^ stress: 2700 genes were up-regulated and 7201 genes were down-regulated (**Figure 3**). Volcano plots and enrichment profiling indicated that these differentially expressed genes (DEGs) were predominantly enriched in “metabolic pathways” and “biosynthesis of secondary metabolites” (ko01100 and ko01110), corroborating that excess Cu^2+^ broadly perturbs primary and specialized metabolism (Bao et al., 2021), thereby impairing normal physiological functions.

Gene Ontology (GO) enrichment further highlighted significant over-representation of molecular functions related to oxidoreductase and transferase activities (**Figure 4**). The concordance between these GO terms and the observed increases in antioxidant enzyme activities substantiates that peanut enhances its ROS-scavenging capacity by transcriptional up-regulation of antioxidant-related genes, thereby improving survival under Cu^2+^ stress (Leng et al., 2015).

The MAPK cascade is a highly conserved signaling pathway that transmits extracellular stimuli through sequential phosphorylation events, thereby modulating gene expression, cell proliferation, differentiation, and programmed cell death (Seger and Krebs, 1995). In plants, this cascade has been shown to orchestrate responses to a wide array of abiotic stresses. For instance, early studies in Arabidopsis revealed that Fe deficiency triggers MAPK-mediated phosphorylation cascades that modulate downstream transcription factors (TFs), thereby initiating defense reactions (Jian et al., 2024). Similarly, in mammalian systems, JNK and p38-MAPKs govern inflammatory and apoptotic responses, whereas the ERK pathway primarily regulates cell growth and differentiation (Moustafa et al., 2014). In our study, the Cu²^+^-induced upregulation of *MPK4* and *MKK2* (**Figure 6**) and the strong upregulation of all 13 DEGs from the LBD and NAC transcription factor families (**Figure 7**) occurred concurrently. This co-expression pattern may suggest that under copper stress, MPK4 protein in peanut roots has the opportunity to phosphorylate members of the LBD and NAC families, thereby activating downstream stress-responsive genes for Cu²^+^ detoxification or adaptation (Guo et al., 2021).

NAC transcription factors are widely recognized for their significant role in improving plant tolerance to abiotic stresses such as drought and salinity (Shu et al., 2024). In recent years, an increasing number of studies have also begun to uncover their new functions in enhancing plant resistance to heavy metal stress. For instance, heterologous overexpression of the *AemNAC2* gene from *Aegilops markgrafii* in wheat significantly enhanced the plant’s tolerance to cadmium stress while markedly reducing the cellular cadmium levels (Du et al., 2020). Similarly, in rice, overexpression of *OsNAC300* increased the tolerance of transgenic rice to cadmium stress, whereas its knockdown resulted in increased sensitivity to the heavy metal (Liu et al., 2023). Furthermore, a study involving the heterologous expression of the *EuNAC9* gene from *Eucommia ulmoides* in yeast demonstrated that it enhanced the yeast’s tolerance to both copper and manganese stress. This was accompanied by the upregulation of the yeast *ScSMF1* and the *ScSOD2* (Zhan et al., 2024). However, direct evidence is still scarce regarding the precise heavy metal-responsive genes whose transcription is regulated by these factors.

Although LBD proteins are widely recognized for their role in regulating lateral organ development (e.g., lateral roots and leaves) (Lee et al., 2009, Lee et al., 2019), some reports have also indicated their function in responding to salt and drought stresses (Guan et al., 2023; Chen et al., 2024). However, their role in enhancing plant heavy metal tolerance is seldom reported. Based on the findings of the present study (**Figure 4** and **Figure 5**), the upregulation of LBD-encoding genes may have a deeper significance. Under copper stress, the plant might actively reduce lateral root growth by upregulating these genes. This strategy is likely aimed at reallocating limited biological resources from root morphological development to more critical defense mechanisms, such as GSH-dependent heavy metal detoxification, POD-mediated antioxidation and cell wall biosynthesis, thereby prioritizing cell survival and homeostasis.

Additionally, although authentic Cu^2+^ receptors remain unidentified, our RNA-seq revealed several DEGs encoding membrane proteins — such as receptor-like kinases (RLKs), wall-associated kinases (WAKLs), LRR-RLKs, and ZIP transporters (**Supplementary Table S3**) — as candidate upstream Cu^2+^-perception components for future validation.

In this study, the MAPK cascade is positioned as a central hub: upstream Cu^2+^ signals (extracellular Cu^2+^ → ROS/NO burst → MAPKKK activation) feed into the module, while downstream phosphorylated MPKs target WRKY, bHLH and ERF transcription factors (**Figure 6**, **7**). RNA-seq identified eight differentially expressed MAPKKKs, one MAPKK and five MPKs, together with 75 TF genes (21 bHLH, 28 ERF, 8 NAC etc.), providing the first transcriptional evidence that this linear “Cu^2+^ → ROS/NO → MAPK → TF → defense gene” axis operates in peanut roots under graded Cu^2+^ stress. The precise molecular interactions among these MAPK components and their target TFs warrant further investigation.

## Conclusions

Based on the integrated physiological and transcriptomic studies, this research definitively demonstrates that the gene response in peanut seedling roots exhibits remarkable specificity within the “Cu²^+^ → ROS/NO → MAPK → TF → defense gene” model. Specifically, we have shown that copper stress strongly induces the expression of *MPK4*, a key nodal point within the MAPK pathway. Post-translationally, MPK4 is highly likely to phosphorylate two critical protein classes: NAC and LBD. Within this unique regulatory network, NAC functions as a core transcription factor, directly regulating the transcription of copper defense-related genes. Concurrently, LBD directly down-regulates genes associated with lateral root growth. This action by LBD, through a reallocation of biological resources, indirectly promotes the increased expression of genes involved in GSH-dependent heavy metal detoxification and secondary oxidative stress (e.g., *GST* and *POD*), thereby cooperatively enhancing the plant’s detoxification and antioxidant capacity.

## Data Availability

The original contributions presented in the study are publicly available. This data can be found at the National Center for Biotechnology Information (NCBI) using accession number PRJNA1311253.
